# Comparison of structural characteristics and bioactivity of *Tricholoma mongolicum* Imai polysaccharides from five extraction methods

**DOI:** 10.3389/fnut.2022.962584

**Published:** 2022-08-05

**Authors:** Nan Zhang, Bing Yang, Kemin Mao, Yuwei Liu, Bimal Chitrakar, Xianghong Wang, Yaxin Sang

**Affiliations:** College of Food Science and Technology, Hebei Agricultural University, Baoding, China

**Keywords:** *Tricholoma mongolicum* Imai, polysaccharide, extraction methods, structure characteristics, antioxidant activity

## Abstract

*Tricholoma mongolicum* Imai is an edible fungus rich in various health-promoting compounds, such as polysaccharides, polypeptides, polyunsaturated fatty acids, etc., and among them, polysaccharides have gotten more attention in recent research trends. This study explored the extraction of polysaccharides from *T. mongolicum* Imai by five extraction methods, including hot water extraction, ultrasound extraction, enzyme-assisted extraction, 0.1 M HCL extraction, and 0.1 M NaOH extraction. The effects of these extraction methods on the yield, chemical structure, apparent morphology, and the antioxidant activities of *Tricholoma mongolicum* Imai polysaccharides (TMIPs) were investigated in this study. The data showed that 0.1 M NaOH extraction produced the highest extraction yield compared to the other extraction methods. The results of high-performance gel permeation chromatography (HPGPC) and scanning electron microscopy (SEM) showed that different extraction methods had significant effects on the molecular weight and morphology of TMIPs. The results of Fourier transform infrared (FT-IR), X-ray diffraction (XRD), and thermogravimetric analysis showed that the extraction methods had no significant difference in functional groups, crystal structure, and thermal stability of TMIPs. The antioxidant activity of TMIPs extracted by ultrasound extraction was more prominent among the five polysaccharides, which might be related to a large number of low-molecular-weight components in molecular weight distribution.

## Introduction

*Tricholoma mongolicum* Imai is a rare edible fungus growing in grasslands. Due to the deteriorating natural environment and over picking, the yield of *T. mongolicum* Imai has extremely reduced, and the producing area is distributed in Xilin Gol League, Hulunbuir League, Inner Mongolia, and Zhangjiakou, Hebei, China. *T. mongolicum* Imai is a world-famous wild edible fungus variety with thick, fragrant, and delicious meat and excellent taste. It is known as the first of the “eight treasures of grass” (1). *T. mongolicum* Imai is rich in polysaccharides, polypeptides, polyunsaturated fatty acids, and other bioactive substances, giving it various functional properties, such as anti-oxidation (2), anti-aging (1), anti-tumor (3), hepatoprotective effects (4), other medical and healthcare regulating human metabolism (5), and enhancing immunity (6). In recent years, there are few studies on the polysaccharides of *T. mongolicum* Imai’s fruiting bodies or spores.

Polysaccharide is a chain polymer formed by a linear or branched connection of aldoses or ketoses through a glycosidic bond, which is the most abundant biopolymer in nature. As one of the important sources of polysaccharides, fungal polysaccharides are widely present in the cell walls of higher macrofungi. Fungal polysaccharides are active substances extracted from true mycelium mycelia and their fermented broth, which have antioxidant (7), anti-aging (8), anti-inflammatory (9), anti-tumor (10), hypoglycemic (11), immunoregulation (12), and other healthcare functions. At present, the commercialized fungal polysaccharides are derived from *Lentinus edodes*, *Polyporus umbellatus*, *Ganoderma lucidum*, *Coriolus versicolor*, tree tongue, etc. In recent years, the research on fungal polysaccharides in the world mainly focused on the molecular mechanism and application of *Lentinus edodes* and *Ganoderma lucidum* polysaccharides as immune activators to enhance immunity and anti-tumor (13, 14), while there were only a few reports on *T. mongolicus* Imai polysaccharides. Generally speaking, the biological activity of polysaccharides is closely related to its structural parameters, including sugar composition, molecular weight, and surface morphology, therefore, revealing that its chemical composition and structure are of great significance for further study of its biological activity. It has been widely accepted that the extraction methods of polysaccharides have an important impact on their structural and physicochemical properties (15).

Polysaccharide extraction methods include hot water-, ultrasound- assisted-, enzyme- treated-, acid- assisted-, alkali-assisted-extraction, and so on. Polysaccharide extraction requires cell wall destruction to improve extraction efficiency. Hot water extraction is generally easy to operate and does not cause polysaccharide degradation. However, the extraction is only from the outside of the cell wall with low extraction rates and longer extraction times (16). Ultrasound-assisted and enzyme-assisted extractions hydrolyze or mechanically destroy the cell wall (17, 18), but the structures of the polysaccharides may also be destroyed by rigorous extraction conditions of ultrasound or the enzymes used. Acid extraction is based on the feature that certain polysaccharides are soluble in dilute hydrochloric acid (19), where the cell wall fully absorbs water, then swells and breaks, thereby releasing polysaccharides and achieving the purpose of extraction. During the extraction process, other components such as protein and phenolic compounds may also be extracted, therefore, the yield of polysaccharides shows a higher amount than the actual value. In alkali-assisted extraction, hydroxyl ions interfere with the hydrogen bond between polysaccharides and release them into the solution to improve the polysaccharide yield (20). During acidic or alkaline extraction, the final extract needs to be neutralized to prevent the degradation of the target polysaccharide.

The above-mentioned extraction methods determine the structural and functional properties of the extracted polysaccharides (15). Therefore, this study aimed to extract the polysaccharides from *T. mongolicum* Imai by five methods, hot water, ultrasonic- and enzyme-assisted extraction, 0.1 M HCl, and 0.1 M NaOH. The antioxidant activity, microstructure, monosaccharide composition, and molecular weight of polysaccharides extracted by the five methods were compared. This study can provide basic data for further extraction, development, and utilization of *T. mongolicum* Imai polysaccharides.

## Materials and methods

### Materials and reagents

Dried fruiting bodies of *T. mongolicum* Imai were obtained from Zhangjiakou, Hebei Province, China. 1,1-Diphenyl-2-picrylhydrazyl (DPPH) was purchased from Solarbio Science and Technology Co., Ltd. (Beijing, China). The total antioxidant capacity assay kit for the ABTS method was purchased from Beyotime Co., Ltd. (Shanghai, China). Monosaccharide standards (mannose, ribose, rhamnose, glucuronic acid, galacturonic acid, *N*-acetyl-D-glucosamine, *N*-acetyl-D- galactosamine, glucose, galactose, xylose, arabinose, and fucose) were purchased from Shanghai Yuanye Bio-Technology Co., Ltd. (Shanghai, China). All the reagents and chemicals used in this study were analytical or HPLC grade.

### Extraction of polysaccharide from *Tricholoma mongolicum* Imai fruiting bodies

#### Pretreatment of *Tricholoma mongolicum* Imai fruiting bodies

The whole dried *T. mongolicum* Imai fruiting bodies were dried in an oven at 60°C for 8 h and then ground into a powder using a kitchen grinder. The powdered *T. mongolicum* Imai was treated with petroleum ether (30–60°) at a ratio of 1:5 (w:v) for 10 h to degrease. Then, the residue after decantation was treated with 75% ethanol at a ratio of 1:5 (w:v) for 4 h to remove the pigmented compounds and small molecular impurities. The residue was decanted and dried at 50°C for 8 h to obtain pretreated dry powder for further use.

#### Hot water extraction

The hot-water extraction of TMIPs was carried out according to the method of (21) with some modifications. Briefly, the pretreated powder of *T. mongolicum* Imai (20 g) was extracted with distilled water (500 ml) at 85°C with continuous stirring for 3 h. Then, the mixture was centrifuged at 4,600 r/min for 15 min to separate the supernatant from the solid residue, which was again extracted following the same procedure two more times. The supernatants were collected and then concentrated to 100 ml by a vacuum rotary evaporator under 55°C. To the concentrated extract, absolute alcohol was added at a 1:3 (v/v) ratio to precipitate the polysaccharides; the precipitate was washed successively with ether, acetone, and n-hexane after filtering, which was freeze-dried to get extracted polysaccharides (Named as TMIPs-H).

#### Ultrasound-assisted extraction

The ultrasound-assisted extraction of TMIPs was carried out according to the method of (22) with some modifications. Briefly, the powder of *T. mongolicum* Imai (20 g) was extracted with distilled water (400 ml) at 60°C for 35 min using a 300 W ultrasonic cleaning apparatus (Geneng, G-100S, Guangzhou). After centrifugation (4,600 r/min, 15 min), absolute ethanol was added at a 1:3 (v/v) ratio to precipitate the polysaccharides; the precipitate was washed successively with ether, acetone, and n-hexane after filtering, which was freeze-dried to get extracted polysaccharides (Named as TMIPs-U).

#### Enzyme-assisted extraction

The enzyme-assisted extraction of TMIPs was carried out by following the method of (23) with slight modifications. Briefly, the powder of *T. mongolicum* Imai (20 g) was extracted with distilled water (500 ml), which was mixed with 1.5% cellulase and 2% pectinase enzymes (Food grade). The pH was maintained at 5 for enzymolysis reaction at 50°C for 100 min. Then, the extraction was carried out in a water bath at 75°C for 4 h. After centrifugation, the supernatant was collected and concentrated to 100 ml in a vacuum rotary evaporator. To the concentrated extract, absolute alcohol was added at a 1:3 (v/v) ratio to precipitate the polysaccharides; the precipitate was washed successively with ether, acetone, and n-hexane after filtering, which was then freeze-dried to get extracted polysaccharides (Named as TMIPs-E).

#### Acid-assisted extraction

The method of (15) was used to extract TMIPs by acid-assisted extraction (15) with some modification. Briefly, the powder of *T. mongolicum* Imai was extracted with HCl (0.1 M) at a 10 ml/g solid–liquid ratio for 1 h; the extraction temperature was maintained at 90°C using a water bath. Then, the pH of the mixture was adjusted to 7.0 and centrifuged at 4,600 r/min for 15 min. The supernatant was concentrated in a vacuum rotary evaporator at 55°C, which was then precipitated with three times the volume of 95% ethanol. The precipitate was washed successively with ether, acetone, and n-hexane after filtering, which was then freeze-dried to get the extracted polysaccharides (Named as TMIPs-Ac).

#### Alkali-assisted extraction

The method was the same as section “Acid-assisted extraction”, except that the extracting solvent was 0.1 M NaOH and the freeze-dried polysaccharide was named TMIPs-Al.

Each sample after freeze-drying was weighed and its yield was calculated according to the following formula:


(1) $$Crudepolysaccharideyield\left(\%\right)=\frac{m}{M}\times 100$$

where, *m* is the mass of dried crude polysaccharide (g) and *M* is the mass of *T. mongolicum* Imai powder (g).

### Chemical composition analysis

Phenol–sulfuric acid method was used to determine the total sugar content (24), and the standard mixture was prepared with glucose. The glucose used was dried to a constant weight in an oven at 60°C. Protein content was measured by the method of Coomassie brilliant blue with bovine serum albumin (BSA) as the standard (25). Contents of uronic acid were analyzed by the 3-phenylphenol colorimetric method with galacturonic acid as the standard (26).

### Molecular weight determination

High-performance liquid gel permeation chromatography (HPGPC) was used to determine the molecular weight distribution of polysaccharides from *T. mongolicum* Imai by using Agilent 1260 high-performance liquid chroma tography, TSK-Gel G4000 SWXL (7.8 mm × 300 mm) gel chromatography column, and refractive index detector (RID). Each dextran standard was dissolved and prepared into a standard solution with a concentration of 4.0 mg/ml, which was then analyzed by high-performance liquid chromatography through a 0.22-μm aqueous phase filtration membrane. Taking the retention time (*x*, min) of each standard as the horizontal axis and the logarithm of the dextran standard molecule (*y*, log M_*W*_) as the vertical axis to fit the regression equation, the corresponding regression equation was *y* = –0.2415*x* + 7.740. The specific detection conditions were as follows: injection volume of 15 μl, column temperature of 30°C, a flow rate of 0.7 ml/min, using ultrapure water as the mobile phase.

### Monosaccharide composition

The monosaccharide composition of *T. mongolicum* Imai polysaccharides was determined by phenyl-3-methyl-5-pyrazolinone (PMP) pre-column derivatization. The 10-ml polysaccharide sample was hydrolyzed with 5.0 ml of 2 mol/L trifluoroacetic acid (TFA) at 120°C for 4 h. After acidolysis, methanol was added and TFA was dried by a nitrogen blowing apparatus. Then 250 μl sample solution was accurately absorbed into a 5-ml Eppendorf (EP) tube, and then 250 μl of 0.6 mol/L NaOH and 500 μl of 0.4 mol/L PMP–methanol were added at 70°C for 1 h. After cooling in cold water for 10 min, 500 μl of 0.3 mol/L HCl was added for neutralization; then, 1 ml of chloroform was added to whirlpool for 1 min and centrifuged at 3,000 r/min for 10 min. The supernatant was carefully taken and extracted 3 times, and the aqueous phase was analyzed by HPLC. The HPLC system consisted of an LC-20AD system equipped with an Agilent ZORBAX Eclipse Xtimate C18 column (5 μm, 4.6 mm × 200 mm) at a temperature of 30°C. The mobile phase consisted of 0.05 M phosphate buffer solution (pH 6.7) and acetonitrile (83:17, v/v) at a flow rate of 1 ml min^–1^, with the sample injection volume of 20 μl. The DAD detector was set at 250 nm to acquire chromatograms. Mannose, ribose, rhamnose, glucuronic acid, galacturonic acid, N-acetyl-glucosamine, glucose, N-acetyl-galactosamine, galactose, xylose, arabinose, and fucose were used as the standards.

### Fourier transform-infrared spectroscopy analysis

A total of 1 mg of each of the dried crude polysaccharides (TMIPs-H, TMIPs-U, TMIPs-E, TMIPs-Ac, and TMIPs-Al) was mixed with 100 mg of dried KBr to prepare the tablet for Fourier transform-infrared (FT-IR) experiment. The IR spectra were recorded at the wavenumber range of 4,000–400 cm^–1^ by a Nicolet FT-IR spectrophotometer (PerkinElmer, Waltham, MA, United States).

### Thermogravimetric analysis

The thermal property of the TMIPs sample was studied using simultaneous thermal analysis (STA2500, NETZSCH, Germany). The heating rate was 10° min^–1^, the heating range was 25–550°C, and the flow rate was 30 ml min^–1^ under a nitrogen atmosphere.

### X-ray diffraction analysis

The dried *T. mongolicum* Imai polysaccharide samples were evenly placed in the sample pool and the sample pool was placed in the X-ray diffractometer (Ultima IV, Neo-Confucianism) operated at 40 kV and 40 mA, while 2θ ranged from 5 to 80°C at a scanning rate of 2°/min.

### Scanning electron microscopy

The surface morphology of each sample was observed by a scanning electron microscope (SEM, ZEISS Gemini 300, Oberkochen, Germany) at an accelerating voltage of 5 kV. Before observation, the dry sample was sputtered with a gold layer, and SEM images were collected by a scanning electron microscope at 500 × and 2,000 × magnification.

### Antioxidant activity

#### Diphenyl-2-picrylhydrazyl radical scavenging activity

The ability of polysaccharide samples to scavenge DPPH free radicals was evaluated according to the previously reported methods of (27). The DPPH solution (1.0 ml; 0.4 mmol/L), prepared with anhydrous ethanol, was mixed with 3.0 ml polysaccharide solution of different concentrations (0.125, 0.5, 1.0, 2.0, 3.0, and 4.0 mg/ml) and place in the dark at room temperature for 30 min. The absorbance of the sample was measured at 517 nm using a UV–Vis spectrophotometer. Ascorbic acid (0.125, 0.5, 1.0, 2.0, 3.0, and 4.0 mg/ml) was used as the positive control. The DPPH scavenging rate was calculated as follows:


(2) $$DPPHradicalsscavengingrate\left(\%\right)=1-\frac{A_{S}-A_{0}}{A_{b}}\times 100$$

where, *A*_*s*_ is the absorbance of the sample group, *A*_0_ is the absorbance value of anhydrous ethanol solution instead of DPPH solution, and *A*_*b*_ is the absorbance value of the polysaccharide sample replaced by deionized water.

#### ABTS radical scavenging activity

The ABTS free radical scavenging of five samples was measured by the readymade kit using ascorbic acid as the positive control. The following formula was used to calculate ABTS free radical scavenging activity of *T. mongolicum* Imai polysaccharides:


(3) $$ABTSradicalsscavengingrate\left(\%\right)1-\frac{A_{s}-A_{0}}{A_{b}}\times 100$$

where, *A*_*s*_ is the absorbance value of polysaccharide sample solution, *A*_0_ is the absorbance value of deionized water instead of ABTS+ solution, and *A*_*b*_ is the absorbance value of deionized water instead of polysaccharide sample.

#### Hydroxyl radical scavenging activity

The assay was performed with a slight modification referring to the method of (28). One milliliter of *T. mongolicum* Imai polysaccharide samples at different concentrations (0.125, 0.5, 1.0, 2.0, 3.0, and 4.0 mg/ml) was taken into the test tubes and the following reagents were successively added: 0.75 mm/L 1,10-phenanthroline (1.0 ml); 0.75 mm/L ferrous sulfate (1.0 ml); 0.01% (v/v) hydrogen peroxide (1.0 ml); and 0.15 mol/L sodium phosphate buffer solution with pH 7.4 (1.5 ml). The mixture was vortex-mixed and incubated at 37°C for 60 min. After the reaction, the absorbance was measured at 536 nm with ascorbic acid (0.125, 0.5, 1.0, 2.0, 3.0, and 4.0 mg/ml) as the positive control and deionized water as the blank control. The hydroxyl radical scavenging rate of the polysaccharide was calculated as follows:


(4) $$Hydroxylradicalsscavengingrate\left(\%\right)=\frac{A_{s}-A_{b}}{A_{0}-A_{b}}\times 100$$

where, *A*_*s*_ is the absorbance of the sample group, *A*_*bji*_ is the absorbance value of the polysaccharide sample replaced by deionized water, *A*_0_ is the absorbance value of deionized water instead of the polysaccharide sample and H_2_O_2_ solution.

#### Statistical analysis

All experiments were repeated three times. The results were expressed as mean ± SD. Every data point was analyzed using SPSS 26 (SPSS, IBM, United States). Duncan’s multiple-range test was used for the analysis of variance. The difference was considered statistically significant when *p* < 0.05 and *p* < 0.01.

## Results and discussion

### Extraction yields and physicochemical properties of *Tricholoma mongolicum* Imai polysaccharides

The yield of the polysaccharide isolated from *T. mongolicum* Imai ranged from 6.64 to 13.16% depending on the extraction method. It can be seen from Table 1 that the output of TMIPs-Al was higher than the rest four methods, which might be due to the destruction and hydrolysis of the cell wall by the alkaline environment, promoting the diffusion of polysaccharides (29). The yield of TMIPs-Ac was the second highest. These results were similar to those reported by (30). Ultrasonic-assisted extraction rate was the lowest, which might be due to the degradation of polysaccharides during ultrasonic treatment, resulting in smaller molecular weight and a decrease in the yield of the polysaccharide during alcohol precipitation process (29). This explanation was further confirmed by the results of molecular weight distribution.

**TABLE 1:** Extraction yield and chemical composition of polysaccharides from *Tricholoma mongolicum* Imai extracted by five different extraction methods.

Item	Sample				
	TMIPs-H	TMIPs-U	TMIPs-E	TMIPs-Ac	TMIPs-Al
Yield (%)	6.64	4.41	5.87	10.83	13.16
Total sugar (%)	36.24 ± 3.44^a^	26.48 ± 0.48^c^	32.96 ± 1.84^a^	29.04 ± 0.56^bc^	32.84 ± 0.04^ab^
Protein (%)	4.93 ± 0.65^d^	4.56 ± 0.27^d^	7.67 ± 0.84^c^	10.18 ± 0.33^b^	16.31 ± 0.18^a^
Uronic acid (%)	3.02 ± 0.82^a^	2.95 ± 0.19^a^	5.13 ± 0.24^b^	2.73 ± 0.13^a^	3.49 ± 0.60^a^
**Monosaccharide composition (molar ratio, %)**					
Mannose	16.80	16.08	15.66	15.03	16.18
ribose	6.30	4.03	4.58	4.50	5.88
Glucuronic acid	1.59	1.72	0.95	0.52	0.19
Glucose	54.65	51.12	57.66	59.75	61.36
Galactose	13.98	20.15	14.63	14.08	9.24
Xylose	2.40	1.65	1.60	1.38	2.16
Fucose	3.50	4.23	3.94	3.72	3.99

Different letters in the same row represent significant differences at p < 0.05.

The total sugar content of the five *T. mongolicum* Imai polysaccharides ranged from 26.48 to 36.24%. The total sugar contents of TMIPs-H, TMIPs-E, and TMIPs-Al were significantly (*p* < 0.05) higher than that of the remaining two polysaccharides. The protein content of TMIPs-Al (16.31%) was significantly higher than the other four polysaccharides of *T. mongolicum* Imai (*p* < 0.05), which was in agreement with the study of (31), where the protein extraction was higher under alkaline condition. The reason might be that the amide bonds in proteins were easily hydrolyzed by the alkali, thus increasing the amounts of protein extracts. TMIPs-E showed the highest uronic acid content (*p* < 0.05), indicating that the uronic acid content of polysaccharides was related to the extraction method.

### Molecular weight of *Tricholoma mongolicum* Imai polysaccharides

**FIGURE 1:**
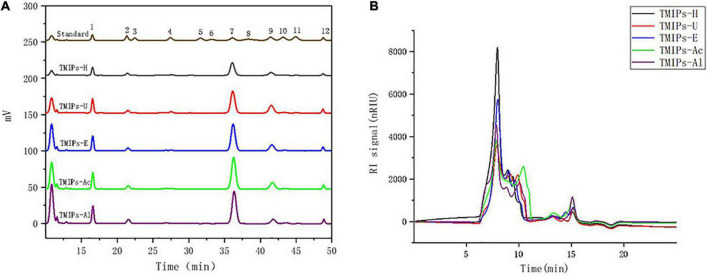
HPLC chromatograms of PMP derivatives of standard monosaccharides and TMIPs **(A)**; 1 – mannose; 2 – ribose; 3 – rhamnose; 4 – glucuronic acid; 5 – galacturonic acid; 6 – N-acetyl-glucosamine; 7 – glucose; 8 – N-acetyl-galactosamine; 9 – galactose; 10 – xylose; 11 – arabinose; 12 – fucose. Molecular weight distribution curve of TMIPs **(B)**.

The biological activity of polysaccharides is affected by molecular weight distribution (32). To analyze the molecular weight distribution of the polysaccharides extracted by these five methods, High-performance gel permeation chromatography (HPGPC) method was used. As shown in Figure 1B, HPGPC spectra of different TMIPs were similar and multiple peaks appeared, indicating that all TMIPs were heteropolysaccharides. Except for TMIPs-Ac, the molecular weight of peak 1 and peak 2 of the other four polysaccharides were similar, ranging from 645.6 to 695.5 kDa and 334.6 to 407.3 kDa, respectively. It can be seen from Table 2 that compared with the molecular weight distribution of TMIPs-H, the proportion of peaks 1 in the molecular weight distribution of TMIPs-U and TMIPs-E decreased and the proportion of peaks 2 and 3 increased, indicating that ultrasound and complex enzyme treatment resulted in an increase in the low molecular weight fraction of the polysaccharides. The reason might be that the polysaccharide was degraded by ultrasound and enzyme to some extent, which converted a part of the high-molecular-weight components into low-molecular-weight components, resulting in the increase of the low-molecular-weight components (29). Similar to our results, Chen and coworkers also proved that the ultrasonic treatment caused the polysaccharides to decompose into smaller molecules (33). The results showed that the molecular weight of TMIPs extracted by alkali and acid extraction was higher than those by hot water extraction, ultrasonic extraction, and enzymic extraction. These findings are consistent with previous reports that ultrasound, complex enzyme, and acid–alkaline treatment can effectively modify the glycosidic bonds (31). In general, the higher the molecular weight of polysaccharides, the greater their tensile strength, impact resistance, and elasticity (34). However, excessively high molecular weights can affect the solubility, rheological properties, processing applications, and even the biological activities of polymers to a certain extent (35). It is reported that polysaccharides with smaller molecular weights usually play a more important role in determining the biological activity than those with larger molecular weights because smaller polysaccharide chains are easier to pass through biofilms and thus can play a more effective role.

**TABLE 2:** Average molecular weight and peak area of different peaks of TMIPs.

Sample	Molecular weight distribution		
	Peak number	Mv (kDa)	Peak area (%)
TMIPs-H	1	657.87	68.63
	2	382.33	11.23
	3	289.69	10.91
TMIPs-U	1	679.20	46.50
	2	334.56	18.55
	3	224.18	19.47
TMIPs-E	1	645.65	57.26
	2	362.05	15.21
	3	260.35	14.77
TMIPs-Ac	1	7,100.35	0.16
	2	692.79	55.77
	3	167.05	27.70
TMIPs-Al	1	695.49	52.39
	2	407.35	11.49
	3	202.83	21.27

### Monosaccharide composition

The monosaccharide in TMIPs samples was identified by comparing the retention times with those of the standards. As shown in Figure 1A and Table 1, *T. mongolicum* Imai polysaccharide was composed of mannose, ribose, glucuronic acid, glucose, galactose, xylose, and fucose. The polysaccharides obtained by the five extraction methods were mainly composed of three monosaccharides, glucose, mannose, and galactose. The molar mass percentage of the three monosaccharides of TMIPs-H was 54.6, 16.8, and 13.9%, respectively, and the molar mass percentage of the three monosaccharides of TMIPs-U was, respectively, 51.1, 16.1, and 20.1%. The molar mass percentage of glucose, mannose, and galactose in TMIPs-E was 57.7, 15.7, and 14.6%. The molar mass percentage of three monosaccharides in TMIPs-Ac was 59.7, 15.0, and 14.1%; the molar mass percentage of the three monosaccharides of TMIPs-Al was 61.4, 16.2, and 9.2%, respectively. These polysaccharides were glucans, which were similar to the results of polysaccharide from *Agrocybe cylindracea* as reported by (36). The composition of monosaccharides had no obvious change, but the molar ratio of monosaccharides was different, which largely depended on the extraction conditions. This finding was consistent with previous reports by (37). The main reason might be that the polysaccharide molecules were partially degraded and the intermolecular hydrogen bond was broken under high temperature and high pressure, ultrasonic or acid-base conditions, resulting in the change in monosaccharide composition. The contents of glucuronic acid and galacturonic acid of the five polysaccharides were low, which was consistent with the results of uronic acid content.

### Fourier transform infrared spectra analysis

**FIGURE 2:**
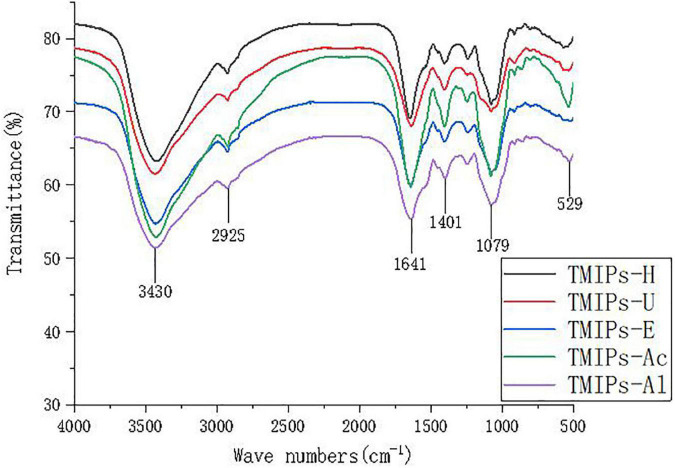
The FT-IR spectrum of TMIPs.

Fourier transform infrared spectroscopy is usually used for the identification of the characteristic functional groups of polysaccharides. As shown in Figure 2, except that some characteristic bands showed the changes in absorbance and wave number, the polysaccharide samples extracted by the five extraction methods had similar general spectral characteristics, confirming the similar chemical compositions of the resultant polysaccharides. Fu and coworkers also proposed that the polysaccharides from loquat (*Eriobotrya japonica*) leaves, prepared by different methods, had similar structures (38). The characteristic peaks of polysaccharides appeared in all the five TMIPs. The broad and intense peak around 3,430 cm^–1^ was observed due to the stretching vibration of the O-H group (39). A weak band around 2,925 cm^–1^ was a characteristic of C-H stretching (40). In addition, the infrared analysis showed that no obvious absorption peak was detected near 1,730 cm^–1^, indicating that the uronic acid in the five polysaccharides from *T. mongolicum* Imai was unesterified uronic acid. The strong peak around 1,640 cm^–1^ was attributed to C=O asymmetric stretching vibration of carboxyl group. The band at about 1,000–1,100 cm^–1^ showed the presence of C-O-C and C-O-H bonds, which was the characteristic peak of pyranose (41). The results of IR spectroscopy indicated that the five extraction methods did not cause damage to the characteristic functional groups and skeletal structures of crude polysaccharides.

### Thermogravimetric analysis and differential scanning calorimetry analysis

**FIGURE 3:**
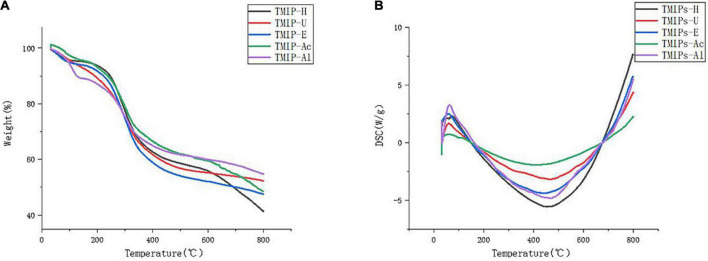
Thermogravimetric analysis of TMIPs. **(A)** TG curve and **(B)** DSC curve.

The thermodynamic properties of polysaccharides were studied by TGA and DSC. TGA curves showed that TMIPs experienced two main mass loss processes. The first mass loss mainly occurred at 25–120°C, which was attributed to the loss of free water in the sample. The second rapid weight loss stage occurred at 575–650°C, which might be related to the thermal decomposition of the polysaccharides (42). Differential scanning calorimetry (DSC) was used to estimate exothermic or endothermic changes with increasing temperature (43). The five polysaccharides exhibited an endothermic behavior at 20–120°C, which indicates that TMIPs are accompanied by the disintegration and mutation of homomorphic configuration during heating and an exothermic behavior at 380–500°C. This difference in thermal stability may be related to the differences in extraction methods, chemical composition, and structure of polysaccharides. Figure 3 shows that polysaccharides from different extraction methods had similar trends in thermodynamic curves, but there are certain differences in their endothermic and exothermic valleys. This difference in thermal stability might be related to the extraction method, chemical composition, and the structure of polysaccharides.

### X-ray diffraction analysis

**FIGURE 4:**
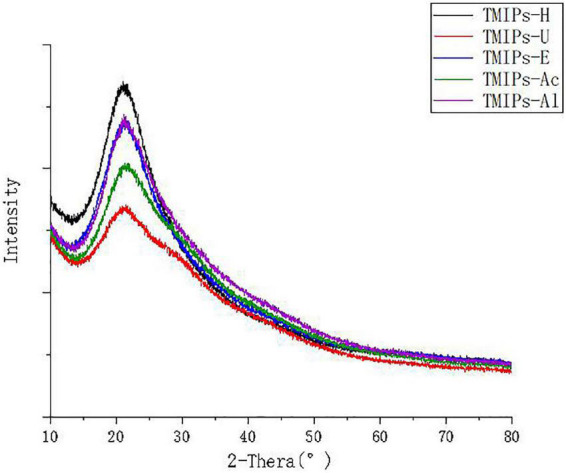
XRD analysis of different polysaccharides from TMIPs.

X-ray diffraction (XRD) is sensitive to the crystal structure analysis of plant polysaccharides. As shown in Figure 4, TMIPs with different intensities showed a single peak of passivation diffraction, when 2θ ranged from 21.17 to 21.47°, indicating that there was an amorphous or semi-crystalline structure inside (44). The XRD patterns displayed the characteristic diffraction curves of polysaccharides. Different extraction methods did not change the crystal structure of *T. mongolicum* Imai polysaccharide significantly.

### Scanning electron microscopy analysis

**FIGURE 5:**
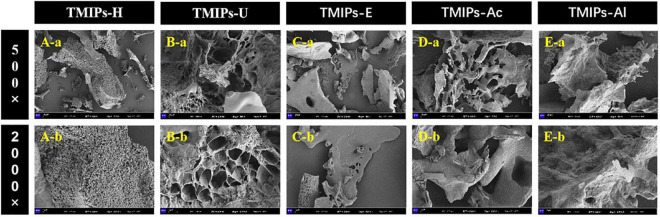
Scanning electron microscopy (SEM) micrographs of TMIPs. **(A)** TMIPS-H; **(B)** TMIPS-U; **(C)** TMIPS-E; **(D)** TMIPS-Ac; **(E)** TMIPS-Al; a. 500 × magnification; b. 2,000 × magnification.

The surface morphology of *T. mongolicum* Imai polysaccharides is displayed in Figure 5 as observed by scanning electron microscope (40). The polysaccharides obtained by different extraction methods had different shapes and sizes in micromorphology (45). TMIPs-H (Figure 5A) showed a massive and rough surface, covered with many small holes and the surface seemed very loose. TMIPs-U (Figure 5B) exhibited a much larger aperture than TMIPs-H and the structure appeared to be more robust with smooth and flat surface (43). This showed that ultrasound might produce acoustic cavitation, causing physical changes. TMIPs-E (Figure 5C) had a large number of small and compact spherical structures. The dense surface morphology of polysaccharides obtained by ultrasound and enzymatic hydrolysis might be attributed to their mild treatment conditions, which didn’t reduce the interaction force and molecular crosslinking degree of polysaccharides completely. Thus, the yield of these two polysaccharides was lower than others. In addition, TMIPs-Ac (Figure 5D) displayed multilayered folds, while the surface of TMIPs-Al (Figure 5E) was rough and uneven, which might be due to the degradation and destruction of cell wall, facilitating the overflow of intracellular substances, improving the extraction efficiency of polysaccharides (37). The results indicated that different extraction methods had obvious influence on the microstructure of TMIPs.

### Antioxidant activities *in vitro*

#### Scavenging activity of *Tricholoma mongolicum* Imai polysaccharides on Diphenyl-2-picrylhydrazyl radical

**FIGURE 6:**
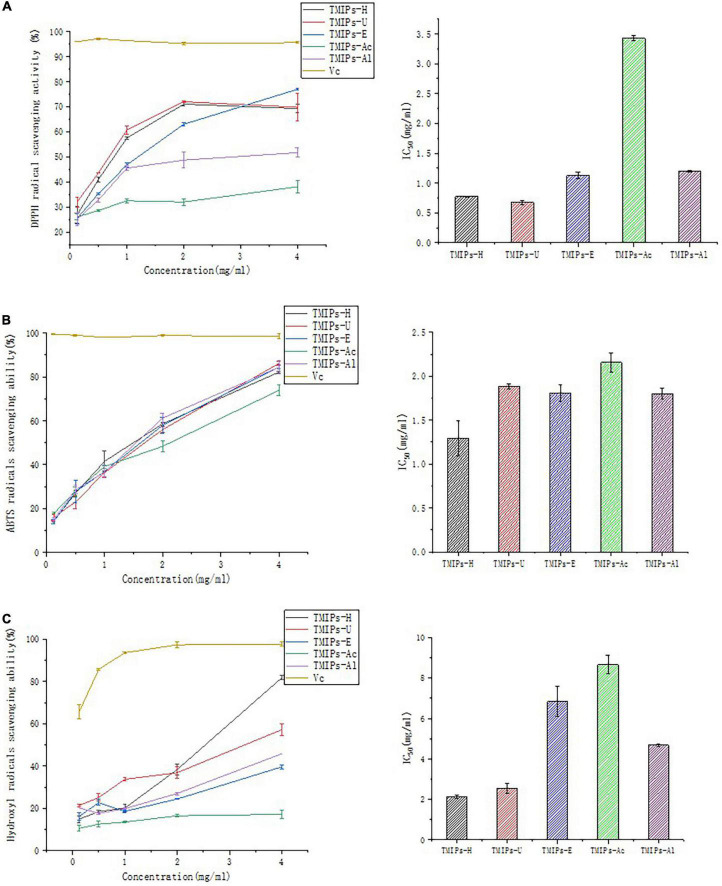
Antioxidant activity of TMIPs. **(A)** DPPH free radical scavenging ability. **(B)** ABTS free radical scavenging ability. **(C)** Hydroxyl radicals scavenging ability.

The DPPH radical scavenging abilities of TMIPs are shown in Figure 6A. The IC_50_ values of TMIPs-H, TMIPs-U, TMIPs-E, TMIPs-Ac, and TMIPs-Al were 0.94, 0.80, 1.27, 3.43, and 1.20 mg/ml, respectively. Ultrasonic extraction showed better DPPH inhibitory activity, which might be attributed to the increase of small molecular weight components of polysaccharides after ultrasonic treatment. Similar results were also reported by Chen and coworkers that low molecular weight usually had high biological activity (46). At the same mass concentration, polysaccharides with lower molecular weight usually have more free hydroxyl groups and higher reducing sugar content, thus having a strong antioxidant capacity. However, the antioxidant activity of polysaccharides is not determined by a single factor, but by comprehensive factors such as chemical composition, molecular weight, and chemical structure (47). The results showed that the antioxidant activity of the polysaccharide extracted by acid and alkali was weak; the reason might be that the internal structure of the polysaccharide was seriously damaged by acid and alkali treatment, which affects their biological activity.

#### Scavenging activity on ABTS radical of *Tricholoma mongolicum* Imai polysaccharides

The free radical scavenging ability of ABTS was often used to evaluate the antioxidant capacity of polysaccharides. It can be seen from Figure 6B that all the *T. mongolicum* Imai polysaccharides showed concentration-dependent scavenging activities. All five polysaccharides exhibited excellent ABTS free radical inhibitory activity. At a concentration of 4 mg/ml, the ABTS radical scavenging rate of TMIPs-H, TMIPs-U, TMIPs-E, TMIPs-Ac, and TMIPs-Al were 82.21, 86.16, 84.51, 73.90, and 84.43 mg/ml, respectively. Although samples’ antioxidant capacities of ABTS were good, they did not exceed those of ascorbic acid. These results indicated that all the extracted TMIPs had the ability to scavenge ABTS free radicals, but the extraction method had an effect on the degree of scavenging activity. These apparent differences in scavenging activity may be related to differences in polysaccharide structure, such as different monosaccharide compositions, which can inhibit the formation of free radicals to varying degrees (31).

#### Scavenging activity of *Tricholoma mongolicum* Imai polysaccharides on hydroxyl radical

The hydroxyl radical is one of the most powerful oxidants and the most active natural free radicals. They disrupt cellular structures closest to the site of their formation and readily attack nucleic acids, proteins, and lipids. Therefore, scavenging hydroxyl radicals is essential to protect living systems (48). As shown in Figure 6C, at a concentration of 0.125–4 mg/ml, the five polysaccharides showed different degrees of antioxidant activity in a concentration-dependent manner. The hydroxyl radical scavenging abilities of TMIPs were similar to their DPPH radical scavenging activities and followed the order TMIPs-H >TMIPs-U > TMIPs-Al > TMIPs-E > TMIPs-Ac. Despite this disparity, these polysaccharides are suitable for application as free radical scavengers. The IC_50_ of TMIPs-H and TMIPs-U were lower than that of the other TMIPs, indicating a better hydroxyl radical scavenging activity, which might be related to a large number of low molecular weight components and different apparent morphologies.

## Conclusion

In this study, the effects of five extraction methods on the chemical composition, structural characteristics, antioxidant activity, and hypoglycemic activity of TMIPs were evaluated. The results showed that all five polysaccharides had typical infrared spectroscopic characteristics of polysaccharides, and different extraction methods had little effect on their crystal structure and thermodynamic properties, showing similar trends. However, the extraction method had effects on its chemical composition, molecular weight distribution, monosaccharide content, and morphology. The yield and protein content of polysaccharides extracted by acid and alkali were higher than other methods. These structural differences led to different biological activities. TMIPs-U had a high DPPH and hydroxyl free radical scavenging capacity.

Studies have shown that different extraction methods affect the chemical composition and structural properties of polysaccharides, which together lead to the pros and cons of their biological activities. The anti-oxidant activity of *T. mongolicum* Imai polysaccharide obtained by ultrasonic and hot water extraction was better, but its extraction rates were lower than that of others. However, ultrasonic extraction can be selected as a more suitable polysaccharide extraction method not only because of the mild extraction conditions but also because the extracted polysaccharide has good biological activity. Therefore, TMIPs-U can be used for mass purification of the polysaccharide in further steps.

## Data availability statement

The raw data supporting the conclusions of this article will be made available by the authors, without undue reservation.

## Author contributions

NZ: conceptualization. NZ, KM, and BY: methodology, writing – original draft preparation, and visualization. BY: software. KM, YL, and BC: validation. BY: formal analysis. KM and XW: investigation. NZ, YL, and YS: resources and supervision. KM, XW, and BY: data curation. NZ, BC, BY, and YS: writing – review and editing. NZ, YL, and YS: project administration. YS: funding acquisition. All authors have read and agreed to the published version of the manuscript.
